# Fluid Supply and Feeding Practices in Cooled Asphyxiated Newborns

**DOI:** 10.3390/children8100899

**Published:** 2021-10-09

**Authors:** Mona Markus, Stamatios Giannakis, Maria Ruhfus, Anja Stein, Axel Heep, Thorsten Plagemann, Peter Jahn, Thomas Hoehn, Ursula Felderhoff-Mueser, Hemmen Sabir

**Affiliations:** 1 Department of General Pediatrics, Neonatology and Pediatric Cardiology, Medical Faculty and University Hospital Duesseldorf, Heinrich-Heine-University Duesseldorf, 40225 Duesseldorf, Germany; mona.markus@hotmail.de (M.M.); stamatios.giannakis@gmail.com (S.G.); Thomas.Hoehn@med.uni-duesseldorf.de (T.H.); 2 Department of Pediatrics I/Neonatology, University Hospital Essen, University Duisburg-Essen, 45147 Essen, Germany; maria.ruhfus@gmail.com (M.R.); Anja.Stein@uk-essen.de (A.S.); Ursula.Felderhoff@uk-essen.de (U.F.-M.); 3 Department of Paediatrics, Elisabeth Children’s Hospital, University of Oldenburg, 26133 Oldenburg, Germany; Heep.Axel@klinikum-oldenburg.de (A.H.); Plagemann.Thorsten@klinikum-oldenburg.de (T.P.); 4 Department of Neonatology, Children’s Hospital Leverkusen, 51375 Leverkusen, Germany; peter.jahn@klinikum-lev.de; 5 Department of Neonatology and Pediatric Intensive Care, Children’s Hospital University of Bonn, 53127 Bonn, Germany; 6 German Centre for Neurodegenerative Diseases (DZNE), 53175 Bonn, Germany

**Keywords:** perinatal asphyxia, hypoxic-ischemic encephalopathy, therapeutic hypothermia, enteral nutrition, parenteral nutrition

## Abstract

Therapeutic hypothermia (TH) for 72 h is the standard treatment to reduce neurological deficits in term newborns with hypoxic-ischemic encephalopathy. There is a large variability regarding nutritional supply during TH treatment in asphyxiated newborns. We performed a retrospective multicentre study in four level I (highest level of care in Germany) NICUs, including 135 asphyxiated term newborns undergoing TH. We analyzed enteral and parenteral nutritional supply during and after TH. We correlated nutritional supply with risk factors for encephalopathy, pH, Sarnat score, mechanical ventilation, seizures, and sedation. A total of 120 of 135 neonates received enteral nutritional supply within the first 24 h, and the majority of children were fully enterally fed within the first 10 days. The grade of encephalopathy and mechanical ventilation had a significant influence on the amount of enteral fluids (*p* = 0.01), whereas the pH and appearance of seizures did not affect the amount of nutritional supply significantly. Furthermore, we did not observe any correlation between enteral intake and abdominal complications such as necrotizing enterocolitis. We observed a large variability of feeding regimes in the four participating NICUs. Early enteral feeding among newborns undergoing TH was performed in each NICU and was well tolerated without increased rates of complications.

## 1. Introduction

Hypoxia-ischemia (HI) following perinatal asphyxia may cause neonatal encephalopathy (NE) in term infants. Clinically NE is associated with the onset of seizures and may lead to short and long-term problems such as multi-organ failure, mental retardation, abnormal feeding, or respiratory distress [1]. Therapeutic hypothermia (TH) for 72 h started within the first 6 h after birth is the treatment of choice reducing death or neurological disabilities in newborns suffering hypoxic-ischemic encephalopathy (HIE) [2].

Due to a lack of evidence, there are no clear guidelines regarding nutritional supply during TH in newborns with HIE following perinatal asphyxia. Enteral and also parenteral nutrition is an important topic in asphyxiated children undergoing TH for 72 h. Enteral nutrition is physiological, and parenteral nutrition increases the risk for infection [3]. A U.K.-wide e-mail survey reports that only 31 percent of neonatal intensive care units (NICUs) follow guidelines concerning nutritional supply during TH [4]. Only 59 percent of the participating hospitals started enteral feeding during cooling [4]. Therefore, there is great variability in terms of feeding regimes. It has been shown that minimal enteral nutrition in cooled asphyxiated newborns is feasible [5]. Furthermore, it was found that minimal enteral nutrition under TH causes a shortened length of hospital stay and time until full oral feeds are achieved [6]. One of the main reasons to withhold enteral feedings during TH is the anxiety for the development of side effects, for example, necrotizing enterocolitis (NEC). In contrast to the proposed understanding that a delay of enteral feeding might prevent NEC, a study from the U.K. reported that a delayed onset of enteral feeding did not reduce the risk of developing NEC in preterm infants [7]. In contrast, it resulted in a delay of time to full enteral feeds [7].

Furthermore, the early introduction of enteral nutrition also bears several advantages. It has a positive effect on gut-barrier function, immune function and reduces bacterial translocation [8]. A delayed start of enteral nutrition results in an increasing rate of chronic lung disease, intestinal inflammation, and an increased risk of morbidity [9]. In addition, both hyperglycemia and hypoglycemia can lead to poor neurological outcomes in cooled asphyxiated newborns [10]. Therefore, it is important to establish an optimal feeding regime for cooled asphyxiated newborns.

In the present study, we collected data from four level I (highest level of neonatal care) NICUs in Germany with regard to enteral and parenteral nutrition in asphyxiated newborns with HIE undergoing TH. The aim was to identify similarities and differences in the nutritional fluid regime in order to recognize possible pros and cons of early initiation of enteral feeding.

## 2. Materials and Methods

### 2.1. Data Collection

Data from 135 term (≥37 + 0 weeks of gestation) neonates born between 2008 and 2019 from 4 level 1 NICUs in Germany were retrospectively collected and analyzed. The study was approved by the local ethical committees. Thirty-eight newborns were born at (*n* = 6) or transferred to (*n* = 32) the NICU of the University Hospital Oldenburg; thirty-nine newborns were born at (*n* = 24) or transferred to (*n* = 15) the NICU of the University Hospital Essen; 38 were born at (*n* = 24) or transferred to (*n* = 14) the NICU of the Children’s Hospital Leverkusen, and 20 were born at (*n* = 11) or transferred to (*n* = 9) the NICU of the University Hospital Duesseldorf. Parts of data from this cohort have been previously published [11,12].

All newborns were treated with whole-body hypothermia for 72 h, started within the first 6 h after birth, maintaining a rectal temperature of 33–34 °C due to NE of presumed HI. Newborns fulfilled the entry criteria for hypothermia therapy as proposed by the large randomized-controlled cooling trials (Apgar score ≤5 and/or ongoing resuscitation at 10 min, abnormal blood gases with a pH < 7.0 or base deficit ≥16 mmol/L as the immediate criteria for perinatal asphyxia, abnormal neurologic examination as the second criterion, and moderate or severe abnormalities on amplitude-integrated encephalography (aEEG) or seizures as the third). The presence of seizures was defined clinically and/or by aEEG. We used the aEEG pattern classification as in previous studies to define abnormal EEG and detection of seizures [13].

Data for gestational age, sex, birthplace (inborn/outborn), birth weight, survival, Apgar scores at 5 and 10 min, need for mechanical ventilation, lowest pH-, base excess- and lactate values before initiation of cooling, degree of encephalopathy before initiation of cooling (Sarnat score), onset and treatment of subclinical or clinical seizures before and during the cooling period were collected. The lowest pH and base excess are defined as the first gas obtained within the first hour after birth. In 90% of cases, it was obtained from cord gas.

Data of enteral and parenteral nutritional supply was collected until newborns received full enteral feeds, and total nutritional supply was collected over a period of 7 days. Enteral nutrition included human breast milk or replacement formula, and parenteral nutrition included all i.v. carbohydrates, amino acids, and fats. The total nutritional intake consisted of all parenteral and enteral nutritional supplies. NICU1 aimed an initial enteral fluid intake of 7 mL/kg/d and raised the fluids by 41% per day during TH. Children in NICU2 started with an enteral fluid intake of 3 mL/kg/d and increased supply daily by 35% during TH. NICU3 started with 10 mL/kg/d and raised enteral fluids by 44% per day, and NICU4 started with an initial enteral fluid intake of 7 mL/kg/d and increased fluids by 34% daily under TH.

The analysis of enteral nutritional supply included time of initiation of enteral feeding in hours, feeding situation at discharge, days of central and peripheral venous access, and weight gain until discharge. Weight gain was defined as a gain of weight per individual day. Data on cumulative morphine dosage and duration of morphine administration was also collected.

Two NICUs with the greatest difference in terms of the amount of nutritional supply were compared. We focused on the comparison between the amount of total, enteral and parenteral nutrition.

Data for total, enteral and parenteral fluid intake were available for all children except those who died within the first 24 h (*n* = 6). No infant was excluded from the study.

### 2.2. Data Analysis

We correlated nutritional fluid intake with the following risk factors: lowest pH within the first hour after birth (<6.8; 6.8–7.0; >7.0), Sarnat scores (Sarnat grade 1, 2, 3), seizures (seizures vs. no seizures) and ventilatory status (mechanical ventilation vs. no mechanical ventilation) within the first week of life. Pearson’s correlation was used to compare each individual parameter against enteral fluid intake.

Data of cumulative morphine doses were collected in mg/kg/d. In two centers, the average cumulative morphine dose for the whole period was documented; in the other two centers, the average for each day was individually calculated. One center used fentanyl instead of morphine.

Total, enteral, and parenteral fluid supply and change in weight were additionally compared between the two centers with the most divergent regime.

SPSS 26 (SPSS, Chicago, IL, USA) was used for statistical analysis. The statistical significance level was 5%. Mann–Whitney U test was used to analyse nonparametric data between the different centers included within the study. Individual repeated measures were analyzed for nutritional intake over time. Descriptive statistics were used for all variables to identify the median and interquartile range as also the mean and standard deviation for continuous variables and the frequency distributions for categorical variables.

## 3. Results

### 3.1. General

We included 135 newborns in our study (*n* = 66 males, *n* = 69 females). Mean gestational age was 39 ± 2 weeks. A total of 65 children were inborn, and 70 were transferred to the respective NICU within the first six hours of life. Median (range) APGAR scores after 1, 5, and 10 min were 1 (0–10), 4 (0–10), and 6 (0–10). A total of 16 of 135 children died. A total of six children died within the first 24 h and eight children within the first week. All descriptive patient data are presented in Table 1.

### 3.2. Total, Enteral, and Parenteral Fluid Supply

**Figure 1:**
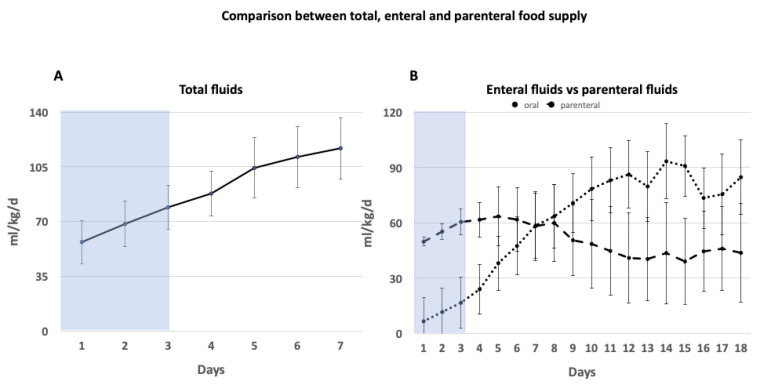
Comparison between total, enteral, and parenteral nutritional supply. Data presented in mean mL/kg/d (+/− SD). Blue shaded box represents time of TH. (72 h). (**A**) Total nutritional fluid intake of the first week of life for children undergoing therapeutic hypothermia given in mL/kg/d. All children received total nutritional supply within the first 7 days and increased supply also during cooling. (**B**) Comparison between enteral and parenteral nutritional supply in children undergoing TH over a period of 18 days given in mL/kg/d.

All NICUs started with a nutritional supply within the first 24 h of life at a mean (±SD) total volume of 55.6 mL/kg/d (±14) and increased the supply daily during cooling by a mean (±SD) of 11 mL/kg/d (±0.5) (Table 2). The largest rise of total nutritional supply was observed after the rewarming phase (day 4 to 5) (mean ± SD: 16 ± 0.75 mL/kg/d). After the fifth day of life, the total nutritional fluid increase was reduced to a mean (±SD) of 6 mL/kg/d (±0.46). After the first week of life, the newborns received twice as much total fluid intake compared to the first day of life (day 7, mean (SD) 113 mL/kg/d (±21.25) (Figure 1A)).

Enteral feeding with human breast milk was started in 120 neonates within the first 24 h (mean (SD) 11 h (6 h)). All centers started enteral feeding within the first 72 h, and the majority of newborns were fully enterally fed within the first 10 days (mean (±SD): 9.2 (±4.4 days)).

Enteral feeding was started with a mean (±SD) enteral volume (EN) of 7 mL/kg/d (±2.5) and increased until full enteral feeds were achieved around day 12 with a mean (±SD) volume of 86 mL/kg/d (±24). The highest amount of enteral nutritional fluid intake was observed after 2 weeks (day 14, mean (SD) 93.42 mL/kg/d (±28)). The amount of enteral feeding exceeded the amount of parenteral feeding (PN) after 8 days (EN: 63.76 mL/kg/d ± 21, PN: 59.96 mL/kg/d ± 17) (Figure 1B).

Newborns received a higher amount of parenteral nutritional supply than enteral nutritional supply within the first week of life. The amount of enteral feeding exceeded the amount of parenteral feeding (PN) after 8 days.

### 3.3. Weight Gain

**Figure 2:**
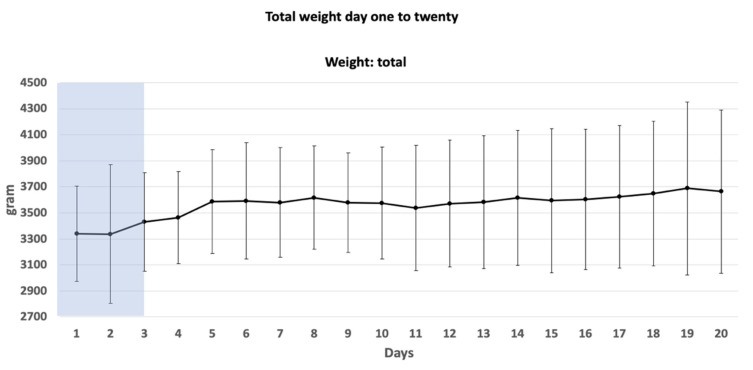
Change in weight of all newborns within the first 20 days after birth. Data presented in mean gram (±SD). Blue shaded box represents time of TH (72 h).

Daily weight gain was analyzed within the first 20 days (Figure 2). A daily weight gain was observed within the analyzed period (mean (SD) weight day 1 vs. day 20 (day 1 3275 g (±420) vs. day 20 3664 g (±627)).

### 3.4. Risk Factors

Newborns with a lower grade of encephalopathy (Sarnat grade 1) received a significantly higher amount of enteral nutrition within the first 6 days compared to children with a higher grade of encephalopathy (Sarnat grade 3) (*p* = 0.01).

**Figure 3:**
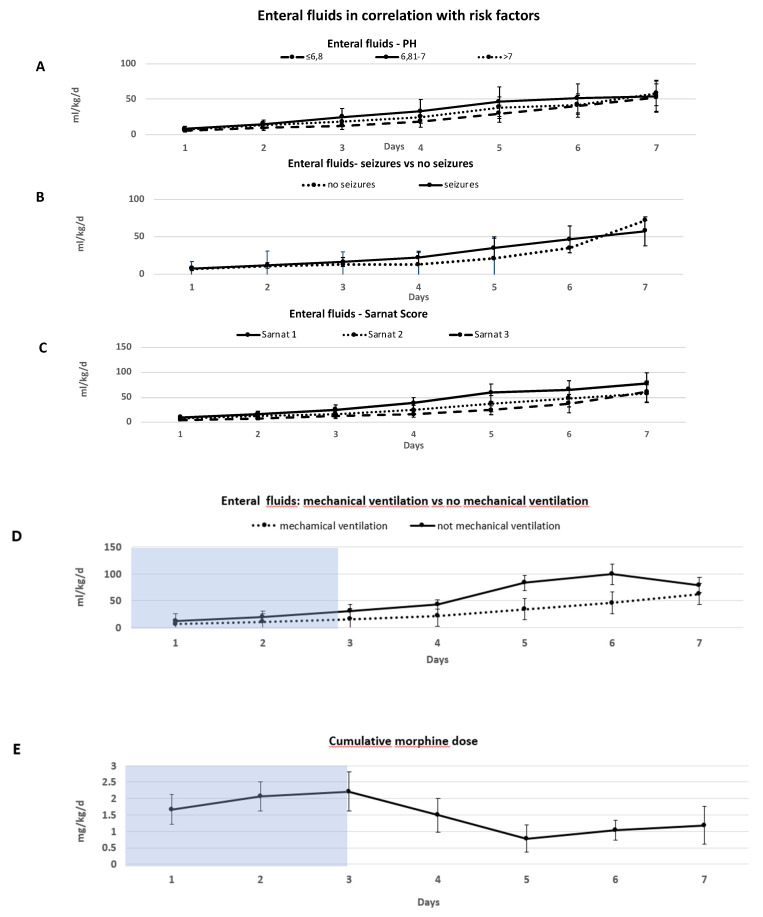
Amount of enteral nutritional fluid supply in the first week of life in correlation with risk factors. Blue shaded box represents time of TH (72 h). (**A**,**B**) Lowest pH after birth (**A**) and appearance of seizures (**B**) did not influence the amount of enteral fluids significantly. (**C**) Grade of encephalopathy (Sarnat score) had a significant influence on the amount of enteral nutritional fluid supply. Neonates with a higher grade of encephalopathy received a significantly lower amount of enteral nutritional fluids. (Sarnat score grade 1 vs. 3 *p* = 0.01, Sarnat grade 2 vs. 3 *p* = 0.02). (**D**) Mechanical ventilation was associated with a significantly lower amount of nutritional intake during the first 6 days (*p* = 0.01) (**D**). (**E**) Cumulative morphine dose over a period of 7 days given in mg/kg/d. After TH was finished (72 h) cumulative morphine dose was strongly decreased. Data presented in mean mg/kg/d (±SD).

Comparing children with Sarnat score grade 2 and 3, children with Sarnat score grade 3 received a significantly lower amount of enteral fluid supply within the first 5 days (*p* = 0.02) (Figure 3C).

Mechanical ventilation was associated with a significantly lower amount of enteral nutritional intake during the first 6 days (*p* = 0.01). With the exception of day 2, newborns without mechanical ventilation received twice as much enteral nutrition in comparison to children with mechanical ventilation in the first 6 days (mean (SD) day 1 and 6 (day 1: 12.2 mL/kg/d (±2.8) and day 6: 99.4 mL/kg/d (±18.7) vs. day 1: 6.1 mL/kg/d (±2.4) and day 6: 45.9 mL/kg/d (±16.1)) (Figure 3D).

First pH after birth and the onset of seizures did not influence nutritional intake significantly (Figure 3A,C).

Comparing the cumulative morphine doses, we observed that the median (range) duration of morphine exposure was 84 (11–288) h (Table 2). Most children were intubated during morphine administration. We did not observe any abdominal complications in children receiving morphine. Morphine was started continuously i.v. at a mean (SD) dose of 0.07 mg/kg/h (±0.0048). During cooling, the mean (SD) morphine dose was increased to 0.09 mg/kg/h (±0.008) and decreased after the end of cooling by a mean (SD) dosage of 0.03 mg/kg/h (±0.008) (day 5) (Figure 3E).

### 3.5. Comparison of the Two NICUs with the Largest Differences

Comparing the two NICUs with the largest differences in amount of total, enteral and parenteral nutritional supply, we observed the following differences: NICU 1 started with a significantly higher amount of total nutritional supply in contrast to NICU 2 (NICU 1 mean (SD) 70 mL/kg/d (±17)) vs. NICU 2 mean (SD) 39.7 mL/kg/d (±7), *p* < 0.005) (Table 3). Furthermore, newborns in NICU 1 received a significantly higher amount of total nutritional supply within the first 4 days (mean NICU1 86.03 mL/kg/d (±14) vs. NICU2 51.53 mL/kg/d (±10), *p* < 0.05).

**Figure 4:**
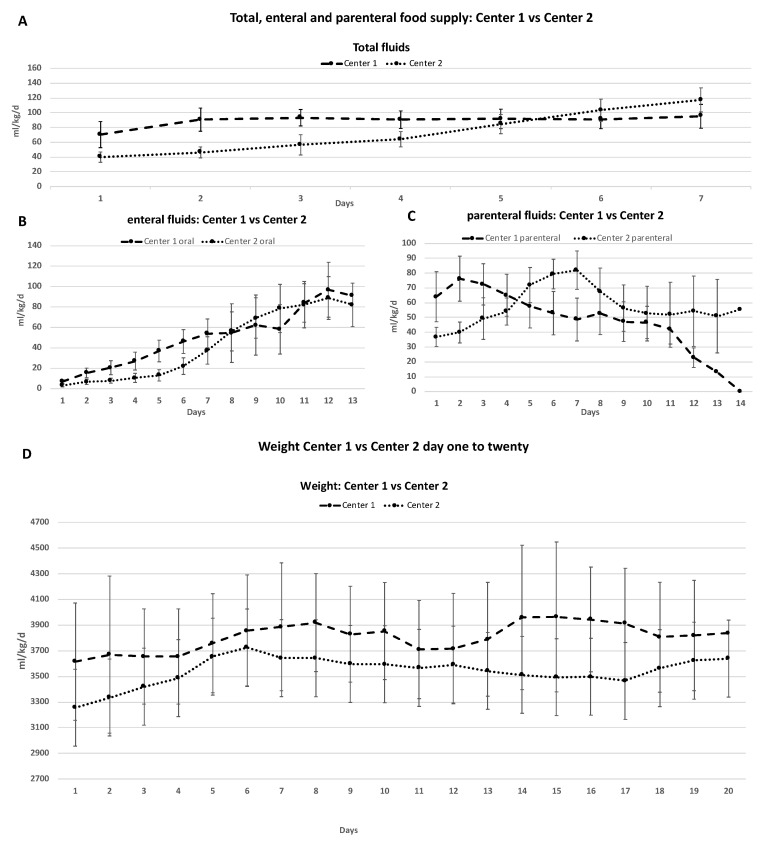
Comparison of total, enteral, and parenteral nutritional supply in two different NICUs. Data presented in mean mL/kg/d (±SD). Blue shaded box represents time of TH (72 h). (**A**–**C**) NICU 1 received a significantly higher amount of total (**A**), enteral (**B**), and parenteral (**C**) nutrition. (**D**) Comparison of change in weight over a period of 20 days. Data presented in mean gram (±SD).

While the nutritional intake in children in NICU 1 only increased until the second day of life and then continuously given between 90 mL/kg/d ± 15 and 95 ± 16 mL/kg/d (minimum, maximum), the nutritional fluid intake for children in NICU 2 increased daily. The increase in newborns in NICU 2 was higher when TH was finished (+8 mL/kg/d vs. +17.7 mL/kg/d) (Figure 4A).

We also observed differences in enteral nutrition. NICU 1 started with a significantly higher amount of enteral nutrition (6.85 mL/kg/d (±1.8) vs. 3 mL/kg/d (±1.3)), and children received a significantly higher amount of enteral nutrition within the first week (*p* < 0.05). During the cooling phase, children in NICU 1 received more than twice the amount of enteral nutrition compared to NICU 2. (NICU 1: 14.23 mL/kg/d (±4.4), NICU 2: 5.76 mL/kg/d (±2.1). NICU 1 showed a lower cumulative morphine dose (0.04 ± 0.02 mg/kg/h), an earlier start of enteral nutrition (11.5 h ± 5.6 vs. 16.4 h ± 22), an earlier timepoint when newborns were fully enterally fed (8 days ± 2.5 vs. 12.8 days ± 4.4) and a shorter period of central venous access (6 days ± 3 vs. 11 days ± 5). Both NICUs showed no increase in the rate of complications such as sepsis or necrotizing enterocolitis (Figure 4B).

Focusing on parenteral nutrition (PN) between the two NICUs, NICU 1 started PN with a significantly higher amount than NICU 2 (NICU1 64.1, ±16.9 mL/kg/d vs. NICU2 36.86, ±6.5 mL/kg/d, *p* = 0.001). Newborns in NICU 1 received a significantly higher amount of parenteral nutrition during the cooling phase and from day five to seven. NICU 1 increased parenteral fluid supply until day 2 (76.22 mL/kg/d, ±15.2) and then decreased parenteral fluid intake until day 14. NICU 2 increased parenteral fluid supply until day 7 (81.97 mL/kg/d, ±12.97) and decreased parenteral fluid supply afterward. (Figure 4C).

### 3.6. Complications during Enteral Feeding

We observed no abdominal complications such as necrotizing enterocolitis in children receiving enteral feeds during and after cooling. Sepsis (defined as positive blood culture) was found in 25 of 135 children; however, we did not observe any correlation to enteral feeding supplies.

### 3.7. Situation at Discharge

A total of 100 children were discharged fully breastfed and 17 (13%) children with a gastric tube. A total of 16 (12%) patients died, and for one patient, the discharge situation was not reported. Breastfeeding was started on day 7 (mean (±2.5) and mean time to full breastfeeding was achieved after 13 days (±5).

## 4. Discussion

This observational multicentre study examined the enteral and parenteral nutritional fluid supply of asphyxiated neonates undergoing TH in four major NICUs in Germany. We found that all neonates started total nutritional supply, and the majority started enteral nutrition within the first 24 h after birth. All NICUs started enteral feeding during the cooling period, within the first 72 h after birth. Different factors such as mechanical ventilation, morphine dose, and grade of encephalopathy had a significant influence on the amount of enteral fluid supply. We did not find any association between enteral feeding and abdominal complications during the cooling period or after rewarming. Comparing the two NICUs with the largest differences in feeding and fluid practices, we observed meaningful differences in amount of total, enteral, parenteral nutritional supply, and morphine dose. This was independent of the level of encephalopathy.

Therapeutic hypothermia (TH) for 72 h is the standard therapy in newborns ≥36 + 0 weeks of gestation suffering from moderate or severe hypoxic-ischemic encephalopathy (HIE) following perinatal asphyxia [14]. TH improves neurologic outcomes at two years, reduces mortality, and does not increase major disabilities [2,14,15].

Enteral and parenteral nutrition is an important aspect during TH, as metabolism may be reduced during TH. Therefore, it is surprising that there are no clear guidelines regarding nutritional supply in cooled asphyxiated newborns. A U.K.-wide e-mail survey reported that only 31% of participating NICUs follow clear guidelines concerning nutritional supply during TH [4]. Our own national guideline in Germany has no recommendation regarding the nutritional supply during cooling [16], and also, guidelines from other countries do not provide clear advice regarding enteral nutrition and recommend a more individual decision for each child [17].

In our study, we found that the nutritional regime in asphyxiated cooled newborns was not standardized in general but that neonates were managed very individually with large differences between different NICUs. The decision process was not always concise, and we were unable to comprehend some of the decisions.

The key reason to withhold enteral nutrition in newborns during TH remains the potential risk of developing NEC. The pathogenesis of NEC is not completely understood. NEC is assumed to arise from multifactorial reasons such as genetics, abnormal microbial colonization, intestinal immaturity, and immunoreactive intestinal mucosa [18]. Based on the belief that enteral feeding is thought to cause NEC, it is often started rather restrictively during TH. On the other hand, enteral nutrition in newborns with breast milk is physiological and has positive effects such as stimulation of the intestinal motility, support of the intestinal immune system, protection of the intestinal mucosa, and stimulation of intestinal hormones.

Despite the risk of developing NEC in our study, enteral feeding was started in 120 of 135 neonates within the first 24 h of life, and all children started enteral feeding during TH (within the first 72 h of life). We did not observe any case of NEC in our cohort. Furthermore, the amount of enteral and also parenteral nutrition was increased in all investigated NICUs during TH. Other NICUs in Europe showed different management in commencing enteral feeding. An e-mail survey from the U.K. reported that almost 60% of participating NICUs started enteral feeding during TH and rewarming [4]. Moreover, a retrospective cohort study from U.K. and Sweden showed that in the Swedish cohort, 91% of newborns were enterally fed during cooling, and in the U.K. cohort, just 31% [5]. Although the beginning of enteral nutrition was managed differently, none of the infants in our study, as well as in the U.K. and Swedish cohort, developed abdominal complications such as NEC [5].

Comparing the two NICUs with the greatest difference in nutritional fluid intake during TH in our study, we found that NICU 1 started with almost twice the amount of total nutritional fluid intake compared to NICU 2. Children in NICU 2 received a significantly higher morphine dose and almost no enteral nutrition during TH. NICU1 with the greater amount of enteral nutrition on day 1 had an earlier start of enteral nutrition and an earlier timepoint until newborns received full enteral feeds. The feeding regime was independent of the level of encephalopathy. Similar outcomes were reported in a study from Florida, which showed that minimal enteral nutrition during TH led to a reduced time until newborns were fully orally fed and a reduced length of hospital stay [6]. Other benefits of enteral nutrition during TH, such as higher survival to discharge, shorter time of parenteral nutrition, and earlier breastfeeding in comparison to children without enteral feeding during TH, were reported [19]. In contrast, a study from U.K. and Sweden reported that delayed enteral feeding did not alter the time to full enteral feeding, and children with delayed enteral feeds had a shorter median length of hospital stay than children with an early onset of enteral nutrition [5].

Although different amounts of enteral and total fluids were given in our study between NICU 1 and 2, we observed no rise in complication rate in either of the NICUs. In addition, a retrospective study from 2021 compared outcomes in infants who were fed versus not fed enterally during therapeutic hypothermia. They showed that the incidence of NEC and late-onset infection was reduced in children receiving feeding during TH [19].

It is also important to note that different factors influence the administration of enteral feedings. The administration was significantly different in severe and mild Sarnat scores, as well as in children with and without mechanical ventilation. The appearance of seizures and the lowest pH value after birth did not significantly influence the amount of enteral fluid supply. However, this might be due to the relatively low sample size in this study. Opioid therapy during TH might also have an impact on the restrictive approach to enteral feeding. Opioids such as morphine were given generously during TH to reduce the newborns’ stress levels and improve the effectiveness of TH [20]. Morphine decreases intestinal motility, inhibits gastric emptying, increases sphincter tone, and causes blockage of peristalsis [21]. It might increase the risk for complications such as NEC or transmigration peritonitis.

In our cohort, it is noticeable that the high dose of morphine during TH was rapidly reduced after TH was finished (day 4) because the children were no longer exposed to the stress of TH. At the same time, enteral feeding was strongly increased (30%) in all NICUs. In our cohort, NICU 1 used a much lower morphine dose and a much higher dose of enteral as well as total nutritional fluid intake compared to NICU 2, which shows that opioid therapy could be a possible reason for a more restrictive enteral feeding.

Although we revealed large differences in the amount of feeding in the different NICUs, it remains clear that all centers fed the neonates during TH without developing NEC or other morbidities, such as sepsis.

The study has several limitations. A major limitation is the relatively small sample size and that we could not differentiate parenteral fluid supply based on its nutritional composition. A second limitation is the retrospective design. There were no existing feeding protocols. Therefore, it was not possible for us to retrace individual decisions of the clinicians regarding the amount of nutritional supply, choice of medication, sedation, etc. A third limitation is that newborns with mild encephalopathies were included in the study. The decision, why newborns with Sarnat 1 scores were cooled, was based on individual clinical decision making. Another limitation was that other factors that we did not investigate, such as liver function, also could have a meaningful influence on the tolerability of enteral nutrition. A final limitation is the missing long-term outcome data, e.g., the Bayley Scales of Infant Development (BSID). Nevertheless, it is important to obtain insight into the similarities and differences of the feeding regimes of four large NICUs in Germany, especially because there are no clear guidelines and the data on feeding regimens is scarce.

## 5. Conclusions

Comparing TH management in four major level I NICUs in Germany, we observed variability in the feeding regime. We conclude that early nutrition and especially early enteral nutrition is possible without increasing the rate of complications during TH. Further prospective studies are needed analyzing the influence of perinatal asphyxia and TH on feeding practices and, therefore, possible gut microbiome composition in cooled asphyxiated newborns.

## Figures and Tables

**Table 1:** Demographic data and perinatal risk factors of the total study cohort (*n* = 135).

	Total (*n* = 135)
Birth weigth (mean ± SD g)	3284 ± 817
Gender (n, % male)	66(49)
Gestational Age ( mean ± SD days)	269 ± 5
APGAR Score 5 min median (range)	4(0–10)
APGAR Score 10 min median (range)	6(0–10)
worst pH (mean ± SD)	6.735 ± 1.2
worst Base Excess (mean ± SD)	17.6 ± 14.6
worst Lactate Level (mean ± SD)	11.6 ± 7.2
HIE Grade before HT (*n* = mild, *n* = moderate, *n* = severe)	31 = mild, 75 = moderate, 29 = severe
Inborn (n, %)	65(48)
Death (n, %)	16(12)

**Table 2:** Nutritional supply data of the total study cohort (*n* = 135).

	Total (*n* = 135)
Start oral feeding (mean ± SD h)	11 ± 12.1
Days until full enteral feeds (mean ± SD days)	9.2 ± 4.4
Cummulative Morphin dosage (mean ± SD mg/kg/h)	0.06 ± 0.05
Duration Morphin (mean ± SD h)	84 ± 38
Days central venous access (mean ± SD)	7 ± 4.8
Days peripheral venous access (mean ± SD)	7 ± 4
Weight gain until discharge (mean ± SD g)	232 ± 273
Feeding situation at dischrge	100 = fully breast fed, 17 = gastric tube, 17 = death, 1 = not assignable

**Table 3:** Descriptive and nutritional supply data from newborns of two different NICUs.

	NICU 1 (*n* = 38)	NICU 2 (*n* = 38)
Birth weight (mean ± SD g)	3615 ± 915	3256 ± 636
Gender (n, % male)	20(54)	21(55)
Gestational Age (mean ± SD days)	279 ± 46	272 ± 22
APGAR Score 5 min median (range)	4 (0–8)	4 (0–10)
APGAR Score 10 min median (range)	6 (0–10)	6 (0–10)
lowest pH (mean ± SD)	6.9 ± 0.23	6.9 ± 0.18
worst Base Excess (mean ± SD)	14.8 ± 10.4	19.7 ± 19
worst Lactate Level (mean ± SD)	9 ± 7	12.1 ± 5.7
HIE Grade before HT (*n* = mild, *n* = moderate, *n* = severe)	10 = mild, 26 = moderate, 2 = severe	6 = mild, 23 = moderate, 9 = severe
Inborn (n, %)	6(16)	24(63)
Death (n, %)	5(13)	3(8)
Start oral feeding (mean ± SD h)	11.5 ± 5.6	16.4 ± 22
Days until full enteral feeds (mean ± SD days)	8 ± 2.5	12.8 ± 4.4
Cumulative Morphin dosage (mean ± SD mg/kg/h)	0.04 ± 0.02	0.12 ± 0.05
Duration Morphin (mean ± SD h)	94 ± 37	92 ± 27
Days central venous access (mean ± SD)	6.5 ± 3.1	11.14 ± 5.13
Days peripheral venous access (mean ± SD)	8 ± 5	9 ± 5
Weight gain until discharge(mean ± SD g)	251 ± 360	306 ± 242
Feeding situation at discharge	27 = fully breast fed, 6 = gastric tube, 5 = death,	33 = fully breast fed, 1 = gastric tube, 3 = dead, 1 = not assignable

## Data Availability

Data can be accessed and is available from the authors.
